# Unrecognized ciliary motility disorders in neutrophilic severe asthma exacerbations

**DOI:** 10.1186/s13223-026-01041-1

**Published:** 2026-05-08

**Authors:** Sarita Thawanaphong, Lucia Gonzalez-Bravo, Katherine Radford, Melanie Kjarsgaard, Carmen Venegas Garrido, Sergey Fedosenko, Terence Ho, Lindsey Dyment, Nadia Suray Tan, Kayla Zhang, Adil Adatia, Manali Mukherjee, Myrna Dolovich, Parameswaran Nair

**Affiliations:** 1 Division of Respirology, Department of Medicine, St Joseph’s Healthcare, McMaster University, Hamilton, ON Canada; 2 Division of Pulmonary and Critical Care Medicine, Department of Medicine, Faculty of Medicine, Chulalongkorn University, King Chulalongkorn Memorial Hospital, The Thai Red Cross Society, Bangkok, Thailand; 3 Firestone Institute for Respiratory Health, St Joseph’s Healthcare, 50 Charlton Avenue East, Hamilton, ON L8N 4A6 Canada

**Keywords:** Airway infection, Asthma exacerbation, Ciliary dyskinesia, Exome sequencing, Nasal nitric oxide, Sputum neutrophils

## Abstract

**Background:**

Airway bacterial infections are frequent in severe asthma and are often under-appreciated as contributors to symptoms and exacerbations. We report our experience using integrative diagnostic methods to identify ciliary motility disorders as contributors to neutrophilic exacerbations in patients with severe asthma.

**Methods:**

Targeted exome sequencing for primary ciliary dyskinesia (PCD) was performed on 52 patients with severe asthma who met predefined criteria (≥ 3 respiratory infections or intense sputum neutrophilia, within a 2-year period, along with evidence of type 2 inflammation, including peripheral or sputum eosinophilia, elevated fractional exhaled nitric oxide (FeNO), or elevated serum immunoglobulin E), and without other obvious immunodeficiencies. A subset of patients with PCD-related gene variant(s) underwent ciliary beat frequency (CBF) analysis from nasal epithelial brushings (*N* = 20 with analyzable data), nasal nitric oxide (nNO) measurement (*N* = 15), and T1/2/17 sputum cytokine assays (*N* = 18).

**Results:**

Among 52 patients (mean age 54.2 ± 15.1 years; 59.6% female), 32(61.5%) had PCD-related gene variants. CBF was reduced in 19/20(95%) patients who underwent motility studies (mean CBF of 8.8 ± 2.7 Hz; normal 14.2 ± 1.0 Hz), and correlated significantly with FEV_1_ (r_s_=0.65, *P* = 0.0017) and inversely with peak sputum neutrophils (r_s_=-0.62, *P* = 0.0097). Subnormal nNO levels (< 250 nL/min) were observed in 12/15(80%) patients; 3/15(20%) were < 77 nL/min (PCD threshold). 5 patients showed FeNO discordance (> 25 ppb). Cytokines associated with inflammasome activation were increased in sputum in majority of patients with PCD-related gene variants. Sputum-guided strategy and 7% hypertonic saline treatment enable significant inhaled corticosteroid doses reduction across the entire population and airway-eosinophilia subgroup (*P* = 0.0026 and *P* = 0.0273). Oral corticosteroid doses were reduced in 6/8(75%), and biologics were not initiated in 26/32(81.3%). FEV_1_ improved by 130 ± 291mL, *P* = 0.0273.

**Conclusions:**

Ciliary dyskinesias are prevalent in patients with severe asthma. Identifying ciliary motility disorders can guide effective treatment and reduce unnecessary biologics use in severe asthma when symptoms are infection-driven. Even variants of uncertain significance in PCD-related genes may be associated with airway infections due to ciliary dysfunction, proven by ciliary beat frequency analysis. nNO could be a useful screening tool, but its optimal cutoff in this population needs further study.

**Supplementary Information:**

The online version contains supplementary material available at 10.1186/s13223-026-01041-1.

## Background

In adults with asthma, 3–6% have severe uncontrolled disease, characterized by poor symptom control and frequent exacerbations despite high-dose inhaled corticosteroid (ICS), long-acting β_2_-agonist (LABA) therapy, and comorbidity management [1, 2]. Personalized treatment is crucial as recurrent exacerbations can lead to lung function decline [3]. Current treatment guidelines generally recommend assessing severe asthma phenotype by identifying characteristics of type 2 (T2) airway inflammation, often characterized by airway and peripheral blood eosinophilia, followed by consideration of add-on biologic therapy [4].

However, severe uncontrolled asthma can arise from a complex interplay of various mechanisms that include airway hyperresponsiveness, mucus plugging, airway remodelling and airway infections characterized by neutrophilic bronchitis. Neutrophilic bronchitis, identified by sputum total cell count (TCC) of ≥ 9.7 × 10^6^ cells/g and sputum neutrophils ≥ 64% [5], often goes underappreciated despite its substantial contribution to asthma exacerbations. Exacerbations associated with sputum neutrophilia, especially when sputum TCC is greater than 25 × 10^6^/g or identified as “intense sputum neutrophilia,” are usually linked to bacterial infections, whether in a T2 or non-T2 inflammatory setting [6–8].

Several mechanisms, including primary and secondary immunodeficiencies [9], heterozygosity of cystic fibrosis transmembrane conductance regulator (CFTR) mutations [10], and ciliary dysfunction, link airway neutrophilia to increased infection susceptibility. Understanding these interconnected pathophysiology is essential to tailor treatments that go beyond rotating antimicrobial courses to reduce exacerbation rates. Motile cilia of airway epithelium play a pivotal role in clearing mucus and trapped pathogens. Classic primary ciliary dyskinesia (PCD) is characterized by year-round, daily productive cough, chronic rhinosinusitis from early life, neonatal respiratory distress, and laterality defects [11, 12]. Without a distinct childhood history, PCD is not typically considered a cause of recurrent infection in adults. However, studies have shown that reducing ciliary beat frequency (CBF) and increasing dyskinesia and immobility indices were more prevalent in severe asthma compared to mild asthma, moderate asthma, and healthy controls [13]. Transcriptomics data from airway epithelial brushing and endobronchial biopsy revealed down-regulation of ciliary-related genes in refractory asthma [14].

Diagnosing PCD is challenging, as no single test serves as a gold standard or detects all forms of ciliary dyskinesia [12, 15, 16]. Clinical exome sequencing has emerged as a valuable tool for detecting rare variants associated with genetic disorders, guiding management and informing hereditary risk [17, 18]. But result interpretation should be incorporated with functionally testing, particularly for variants of uncertain significance (VUS). Because PCD is typically autosomal recessive, heterozygous findings need careful correlation. Nasal nitric oxide (nNO) testing, where low values suggest ciliary dysfunction, has high sensitivity and specificity in PCD diagnosis and is widely used as a screening and/or additional test [11, 16, 19]. However, in patients with severe asthma and elevated fractional exhaled nitric oxide (FeNO), the diagnostic role of nNO and the optimal cut-point warrants further study.

The primary objective of this study was to report our experience of identifying ciliary motility disorders as contributors to neutrophilic exacerbations in adult patients with severe asthma referred to a tertiary university clinic for consideration of initiation or switching biologics, employing exome sequencing in blood, integrated with ciliary beat analysis and nNO measurement. The secondary objective is to report the clinical relevance of PCD-related gene variants, identified as variants of uncertain significance, in terms of ciliary beat analysis and clinical presentations and the value of nNO, FeNO, and airway inflammation in patients with severe asthma through sputum biomarkers, including cellular analysis and cytokine profile.

## Methods

We conducted a retrospective cohort study, reviewing the patients’ data in our adult Complex Airway Diseases Clinic of the Firestone Institute for Respiratory Health of St Joseph’s Healthcare (Hamilton, ON, Canada) from October 2018 to April 2025. All the patients with previously physician-diagnosed severe asthma [20] who were referred to our clinic for an appropriate treatment adjustment, especially considering commencing or switching biologics and meeting our clinical criteria to suspect a susceptibility to recurrent airway infections, were included in the study. The criteria to initiate additional tests were patients who presented with ≥ 3 respiratory infections or intense sputum neutrophilia, defined as a TCC of ≥ 25ⅹ10^6^ cells/g and neutrophil > 65% [21], within a 2-year period, and evidence of a T2 inflammation, including peripheral eosinophilia (absolute eosinophils ≥ 0.3ⅹ10^9^/L [22]), sputum eosinophilia (sputum eosinophils > 3% [21]), raised FeNO (> 25 parts per billion (ppb) [23]), or raised serum immunoglobulin E, without another obvious cause of infection, including, CFTR mutation and decreased serum total immunoglobulins or IgG sub-types.

The patients’ demographic data, clinical features, and investigation results were obtained via our hospital’s electronic medical record. All pulmonary function tests, including pre- and post-bronchodilator spirometry, methacholine challenge test and FeNO, were conducted according to standard guideline recommendations [23–25]. Sputum cell analysis data were collected from both spontaneous and induced sputum using hypertonic saline nebulization as previously described [21, 26]. Sputum cytokines (Interleukin (IL)−12p70, IL-15, IL-1β, IL-18, Interferon-*γ*, tumor necrosis factor (TNF)-⍺, IL-6, IL-17A, B-cell activating factor (BAFF), IL-33, IL-4, IL-13, IL-5, and IL-10) were measured using an automated enzyme-linked immunosorbent assay (ELISA) platform (Ella™, ProteinSimple, R&D Systems, Bio-Techne, Minneapolis), with sputum supernatant diluted 1:2 per routine Ella protocol. Thymic stromal lymphopoietin was not included in the panel. Reference ranges were derived from the 90th percentile of healthy controls [27]. In the subset of patients with PCD-related gene variant, ciliary beat frequency analysis and nNO were performed using the technique described below. All tests were done as part of routine clinical care. All data were recorded in electronic case record forms without identifiers.

### Exome sequencing methods

Targeted exome sequencing was performed using Blueprint Genetics PCD and Primary immunodeficiency (PID) panel (Seattle, WA; test code IM0801), an extended panel of 47 PCD-related genes, including non-coding variants [11, 28]. Informed consent was obtained, and peripheral blood was collected for deoxyribonucleic acid extraction, quantification, and fragmentation. Libraries were prepared by adapter ligation, bead-based size selection, and polymerase chain reaction amplification. Regions of interest were hybridization capture and sequenced on an Illumina platform. The sequencing data were mapped to the human reference genome. Variants were classified as pathogenic, likely pathogenic, or VUS, according to Blueprint Genetics Variant classification schemes, modified from the American College of Medical Genetics and Genomics (ACMG) guideline 2015 [29].

### Ciliary beat frequency analysis

Nasal epithelial brushings were collected from a subset of patients with PCD-related gene variants. Sampling was based on test availability during appropriate in-person follow-up, without preferential selection based on clinical severity or genetic results. The procedure was deferred for ≥ 6 weeks post-respiratory infection to mitigate infection-related CBF reduction. Regular treatments, including β₂-agonist and hypertonic saline (HTS) nebulization, were continued, although they may increase CBF. Samples from both nostrils (inferior turbinate, no anesthesia) were obtained separately and suspended in 5 mL Earle’s Balanced Salt Solution. Videos of ciliated cells were prepared using high-speed digital imaging in a temperature-controlled chamber set at 37 °C [30], recorded for ≥ 3 s, and subsequently analyzed using ProAnalyst video imaging processing software, version 8 (XCitex, Cambridge, MA, USA). The mean of CBF from all cells was reported. Ciliary movement pattern was described as “normal, not beating, rotating, beating stiffly, not bending, incomplete stroke, or non-metachronal”. Given that different CBF measurement methods are not interchangeable [31], we use a normal cut-off value of beat frequency of 14.2 ± 1.0 Hz from our laboratory data [30].

### Nasal nitric oxide measurement

nNO measurement was performed using the portable electrochemical analyzer device (NIOX VERO) with the exhalation against resistance technique (30 s) and a sample gas flow rate of 0.3 L/min. All tests were performed by a single operator during a follow-up visit under appropriate conditions (no acute viral respiratory infection, severe nasal obstruction, nasal bleeding, or recent sinonasal surgery). All nasal medications, including nasal rinses, were withheld on the day of testing. Each nostril was tested until achieving 10% intra-nasal and inter-nostril repeatability. The final nNO was the highest value from either nostril, reported in standardized value in nL/min. An nNO cutoff of 250 nL/min was used, based on prior evidence showing that healthy individuals aged ≥ 5 years typically have nNO values above 250–300 nL/min [32, 33].

### Statistical methods

Baseline characteristics were summarized by variable type and distribution. Normality was assessed with the Shapiro-Wilk test. Normally distributed data were presented as mean (standard deviation) (SD)), and non-normally distributed data as median (interquartile range (IQR)). Categorical variables are N(%). Between-group comparisons used unpaired t-test or Mann–Whitney U test; within-subject (pre/post) comparisons used paired t-test or Wilcoxon signed-rank test. Categorical data were compared by χ² or Fisher’s exact test (expected cell counts were < 5). Associations were evaluated using Spearman’s rank correlation (r_s_). *P* values were adjusted for multiple comparisons using the two-stage linear step-up method of Benjamini, Krieger, and Yekutieli (FDR control). All statistical analyses were performed using GraphPad Prism Version 10.5.0; significance was defined as a *P* value < 0.05.

## Results

From October 2018 to April 2025, 542 patients were referred with the diagnosis of severe asthma [20]. Among them, 301 patients exhibited ≥ 1 episode of neutrophilic bronchitis. Of these, 52 patients (9.6% of the severe asthma group and 17.3% of those with neutrophilic bronchitis) met our clinical criteria of ≥ 3 infections or intense sputum neutrophilia, within a 2-year period, with previous evidence of T2 inflammation (absolute blood eosinophils ≥ 0.3 × 10^9^/L, sputum eosinophils > 3%, FeNO > 25 ppb, or raised serum immunoglobulin E) for initiating exome sequencing for the PCD/PID panel. The 52 patients had a mean age of 54.2 ± 15.1 years, with 59.6% female. Peripheral blood eosinophilia was present in 42(80.8%) patients, and airway eosinophilia in 25(48.1%). All patients with airway eosinophilia also demonstrated peripheral eosinophilia. 10(19.2%) patients exhibited combined peripheral and airway eosinophilia with concomitantly elevated FeNO.

**Fig. 1:**
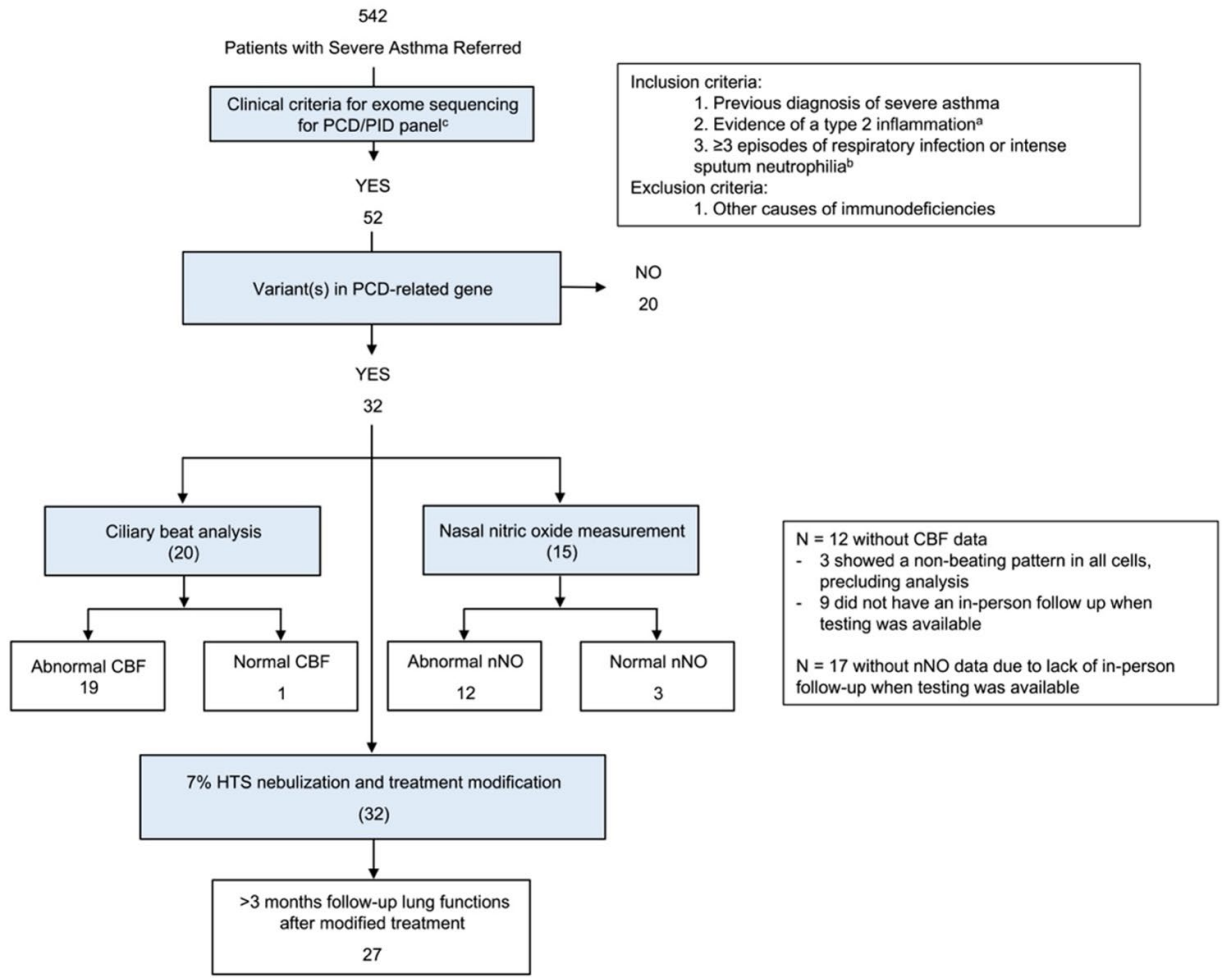
Flow diagram showing the number of patients identified at each step of inclusion/exclusion. CBF = Ciliary beat frequency; HTS = Hypertonic saline; nNO = Nasal nitric oxide; PCD = Primary ciliary dyskinesia; PID = Primary immunodeficiency. ^a^Evidence of type 2 inflammation: Peripheral eosinophilia, sputum eosinophilia, raised FeNO, or raised serum immunoglobulin E. ^b^Intense sputum neutrophilia: Total cell count ≥ 25 × 10^6^ cells/g and neutrophils > 65%. ^c^Blueprint Genetics, Seattle, WA, test code IM0801

The genetic testing revealed PCD-related gene variant(s) in 32(61.5%) patients (Fig. 1). This represented 5.9% of all patients with severe asthma in our cohort who underwent genetic testing based on predefined clinical criteria. Baseline demographics, clinical characteristics, and investigation data are detailed in Table 1. There were no significant between-group differences in concurrent respiratory diagnoses, lung functions, or sputum profile. However, we observed a higher prevalence of nasal polyposis among patients with chronic rhinosinusitis in the variant-positive (PCD-related genes) group compared to the variant-negative group (82% Versus 25%, *P* = 0.0237). Bronchiectasis on chest computed tomography was observed on chest computer tomography at similar rates in the variant-positive and variant-negative groups, 15/32 (46.9%) Versus 11/20 (55.0%), respectively; *P* = 0.5686).

**Table 1:** Baseline participant demographic, clinical characteristics, and investigations

Baseline characteristics	Variant-positive Group (*N* = 32, 61.5%)	Variant-negative Group (*N* = 20, 38.5%)	*P* Value
Age (year)^a^	52.9 (17.0)	56.3 (11.5)	0.4021
Female sex, n (%)	19 (59.4)	12 (60.0)	0.9644
Ex-smoker, n (%)	13 (40.6)	6 (30.0)	0.4389
Current smoker, n (%)	1 (3.1)	1 (5.0)	> 0.9999
*Associated respiratory conditions*			
COPD, n (%)	5 (15.6)	1 (5.0)	0.3867
Bronchiectasis, n (%)	15 (46.9)	11 (55.0)	0.5686
Chronic rhinosinusitis, n (%)	11 (34.4)	8 (40.0)	0.6819
Nasal polyposis, n (%)^b^	9 (28.1)	2 (10.0)	0.1697
AERD	2 (6.3)	0 (0)	0.5173
*Clinical History* ^c^			
Recurrent pulmonary infection, n (%)	27 (84.4)	15 (75.0)	0.4040
Recurrent sinus infection, n (%)	3 (9.4)	3 (15.0)	0.6644
Number of exacerbations (per year)	3 (2.0)	3 (2.0)	0.7802
*Pulmonary function tests*			
FEV_1_ (L)	1.68 (1.3)	1.81 (1.6)	0.8358
FEV_1_ (%predicted)	61.00 (41.3)	71.00 (37.5)	0.7062
FEV_1_/FVC < 70%, n (%)	20 (62.5)	13 (65.0)	0.8555
FeNO (ppb)^d^	20.00 (15.0)	16.50 (8.8)	0.1535
*Sputum analysis* ^e^			
Peak of sputum total cell count (10^6^ cells/g)	29.30 (40.1)	50.00 (67.1)	0.1498
Peak of sputum neutrophils (%)	89.15 (10.1)	96.30 (22.5)	0.0613
Peak of sputum eosinophils (%)	6.25 (13.1)	2.80 (2.9)	0.0930
Patient with evidence of intense sputum neutrophilia, n (%)	15 (53.6)	14 (82.4)	0.0623
Patient with evidence of sputum eosinophilia, n (%)	18 (64.3)	7 (41.2)	0.1304
*Laboratory investigations*			
Peak of blood eosinophils (10^9^/L)	0.6 (0.5)	0.4 (0.6)	0.6228
*Treatments*			
Medium dose ICS/LABA, n (%)	5 (15.6)	3 (15.0)	> 0.9999
High dose ICS or ICS/LABA, n (%)	24 (75)	14 (70.0)	0.6925
ICS dose (mcg/day of FP)	1000 (375)	1000 (500)	0.4046
OCS, n (%)	8 (25.0)	7 (35.0)	0.4387
OCS dose (mg/day of prednisone)^a^	9.81 (5.1)	8.00 (6.1)	0.5478
Biologics, n (%)	4 (12.5)	7 (30.0)	0.0815

Data are presented as No. of patients (%) or median (interquartile range) unless otherwise indicated. COPD = chronic obstructive pulmonary disease; FEV_1_ = forced expiratory volume in 1 s; FP = fluticasone propionate; FVC = forced vital capacity; FeNO = fractional exhaled nitric oxide; ICS = inhaled corticosteroids; LABA = long-acting beta 2 agonist; OCS = oral corticosteroids; ppb = parts per billion.

^a^Data are presented as mean (SD)

^b^Prevalence of nasal polyposis among patients with chronic rhinosinusitis was significantly higher in the variant-positive group compared to the variant-negative group (*P* = 0.0237)

^c^None of the patients had a laterality defect; one female with a PCD-related gene variant reported infertility of unclear etiology

^d^Data from 25 patients in the variant-positive group and 10 patients in the variant-negative group

^e^Data from 28 patients in the variant-positive group and 17 patients in the variant-negative group

Table 2 details the variants in PCD-related genes identified through exome sequencing. Across the cohort, we identified 42 variants in 19 genes: 3 pathogenic, 6 likely pathogenic, and 33 VUS. One patient exhibited homozygosity, while the others were heterozygous. The most common genes found were *DNAH11* (9/32), *DNAH5* (5/32), and *DNAH9* (5/32).

**Table 2:** Primary ciliary dyskinesia panel exome sequencing results

No.	Gene	DNA variation	Predicted effects	Variant type	Classification^a^
			(Protein variation)		
1	*DNAH5*	c.1852 C > T	p.(Arg618*)	Premature protein termination	A
2	*DNAH5*	c.13458dup	p.(Asn4487*)	Frameshift	A
3	*CCDC40*	c.248del	p.(Ala83Valfs*84)	Frameshift	A
	*RSPH4A*	c.1031T > C	p.(Leu344Pro)	Missense	C
4	*CCDC40*	c.248del	p.(Ala83Valfs*84)	frameshift	A
	*DNAH11*	c.3040 C > G	p.(gln1014Glu)	Missense	C
5	*DNAH9*	c.308dup	p.(Leu104Profs*45)	Frameshift	B
6	*ARMC4*	c.183_184del	p.(Ser62Phefs*7)	Frameshift	B
7	*DNAAF5*	c.1499G > T	p.(Cys500Phe)	Missense	B
8^b^	*RSPH9*	c.67G > a	p.(trp226*)	Stop gained	B
9	*DNAH11*	c.6565 C > T	p.(Arg2189*)	Stop gained	B
	*DNAH11*	c.5434G > A	p.(Val1812Met)	Missense	C
	*CCDC65*	c.773T > C	p.(Ile258Thr)	Missense	C
10	*DNAH9*	c.4918 C > T	p.(Arg1640*)	Stop gained	B
	*DNAH5*	c.8921G > A	p.(Gly2974Asp)	Missense	C
11	*DNAH9*	c.28 C > T	p.(Leu10Phe)	Missense	C
	*DNAH11*	c.2435 A > T	p.(Tyr812Phe)	Missense	C
12	*DNAH5*	c.5648G > A	p.(Arg1883Gln)	Missense	C
	*RSPH4A*	c.1082G > A	p. (Gly361Asp)	Missense	C
13	*DNAAF5*	c.829 C > T	p.(Arg277Cys)	Missense	C
	*NME8*	c.1097 A > G	p.(Glu366Gly)	Missense	C
14	*DNAAF3*	c.1615G > A	p.(GLy539Arg)	Missense	C
	*DNAH11*	c.8547G > A	p.(Met2489Ile)	Missense	C
15	*CCDC114*	c.905 A > G	p.(Asn302Ser)	Missense	C
	*LRRC56*	c.129 C > G	p.9Asp43Glu)	Missense	C
16	*HYDIN*	c.11269G > C	p.(Val3757Leu)	Missense	C
	*HYDIN*	c.11,074 A > G	p.(lle3692Val)	Missense	C
17	*CCDC40*	c.170 C > T	p.(Thr57Ile)	Missense	C
18	*CCDC114*	c.880G > A	p.(glu294Lys)	Missense, Splice_region	C
19	*CCDC151*	c.1591–9 C > A	-	Intron	C
20	*CCDC151*	c.1606G > A	p.(Glu536Lys)	Missense	C
21	*DNAAF2*	c.48 C > A	p.(Ser16Arg)	Missense	C
22	*DNAAF5*	c.642G > T	p.(Gln214His)	Missense	C
23	*DNAH5*	c.10,367 C > T	p.(Ala3456Val)	Missense	C
24	*DNAH8*	c.9706 A > G	p.(Met3236Val)	Missense	C
25	*DNAH9*	c.8830 C > T	p.(Arg2944Trp)	Missense	C
26	*DNAH11*	c.11021T > C	p.(Leu3674Ser)	Amino acid substitution	C
27	*DNAH11*	c.13421_13453del	p.(Gln4474_Lys4484del)	Inframe_deletion	C
28	*DNAH11*	c.7055 A > T	p.(Lys2352Ile)	Missense	C
29	*DNAH11*	c.7021T > C	p.(Ser2341Pro)	Missense	C
30	*DRC1*	c.421T > G	p.(Trp141Gly)	Missense	C
31	*OFD1*	c.1616T > C	p.(Val539Ala)	Missense	C
32	*DNAH9*	c.12,495 C > G	p.(Ile4165Met)	Missense	C

These PCD-related gene variants are heterozygosities except for patient No.8, who has homozygosity

^a^Classification of variants: A = pathogenic variant, B = likely pathogenic variant, C = variant of uncertain significance (VUS)

^b^The patient has a homozygous variant

Nasal brushing for CBF analysis was performed in 23 patients with PCD-related gene variant(s). In 3 patients, a non-beating pattern was observed in all cells, precluding analysis. Among the remaining 20 patients, low CBF was identified in 19 patients (95%), with a mean of 8.8 ± 2.7 Hz (normal cut-off value 14 ± 1.0 Hz). Non-beating cells were found in 13 patients, with immobility index 3.2%−85.7% (mean 27.5 ± 24.2). Regarding movement patterns, we identified abnormalities in 7 patients, including stiff beating, rotating patterns, non-bending patterns, and non-metachronal patterns. The details of each patient’s ciliary analysis data are presented in Table 3.

**Table 3:** Ciliary beat frequency analysis data

No. of patient^a^	Mean CBF (Hz)	SEM	Immotility index (%)	Abnormal pattern
1	9.06	1.16	20.00	Beating stiffly
4	9.68	0.9	0.00	Rotating
5	10.56	0.58	14.29	Normal pattern
6	6.25	0.59	38.10	Normal pattern
7	7.77	0.48	3.23	Normal pattern
9	11.19	0.56	0.00	Beating stiffly, Not bending
10	8.39	0.44	14.29	Beating stiffly, Rotating
11	7.25	0.75	34.78	Normal pattern
13	2.26	0.98	55.56	Non bending
14	14.50	1.26	0.00	Normal pattern
17	11.02	0.43	0.00	Nonmetachronal, not bending
18	4.63	0.82	85.71	Normal pattern
19	9.49	0.39	4.44	Normal pattern
20	9.68	1.09	5.26	Normal pattern
23	11.39	0.88	0.00	Normal pattern
25	7.14	5.67	40.00	Normal pattern
26	7.10	0.72	0.00	Beating stiffly
28	10.86	0.56	0.00	Normal pattern
29	7.57	0.9	34.69	Normal pattern
30	10.53	0.59	6.67	Normal pattern

CBF = Ciliary beat frequency; Hz = Hertz; SEM = standard error of the mean

^a^Referred to the number of patients with PCD-related gene variant(s) in Table 2

**Fig. 2:**
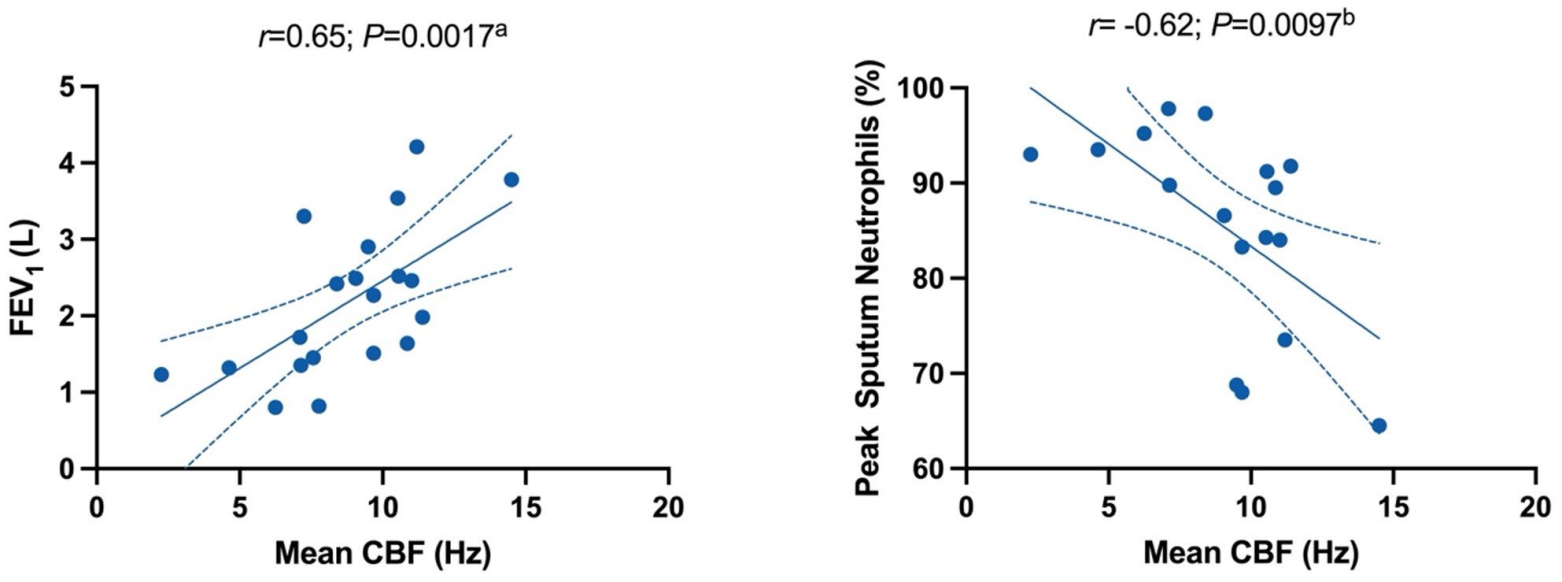
Spearman correlation analysis between mean CBF and FEV_1_ and between mean CBF and peak sputum neutrophils. CBF = Ciliary beat frequency; FEV_1_ = Forced Expiratory Volume in 1 s. ^a^q = 0.0120. ^b^q = 0.0343

We observed a significant correlation between CBF and baseline forced expiratory volume in 1 s (FEV_1_) (r_s_=0.65, *P* = 0.0017, q = 0.0120), as well as with peak sputum neutrophil count (r_s_ =−0.62, *P* = 0.0097, q = 0.0343) (Fig. 2). However, no significant correlations were found between CBF and forced vital capacity (FVC), FeNO, peak sputum TCC, or peak sputum eosinophils. In the eosinophilic-airway subgroup, neither CBF nor immobility index correlated with peak blood or sputum eosinophils.

**Fig. 3:**
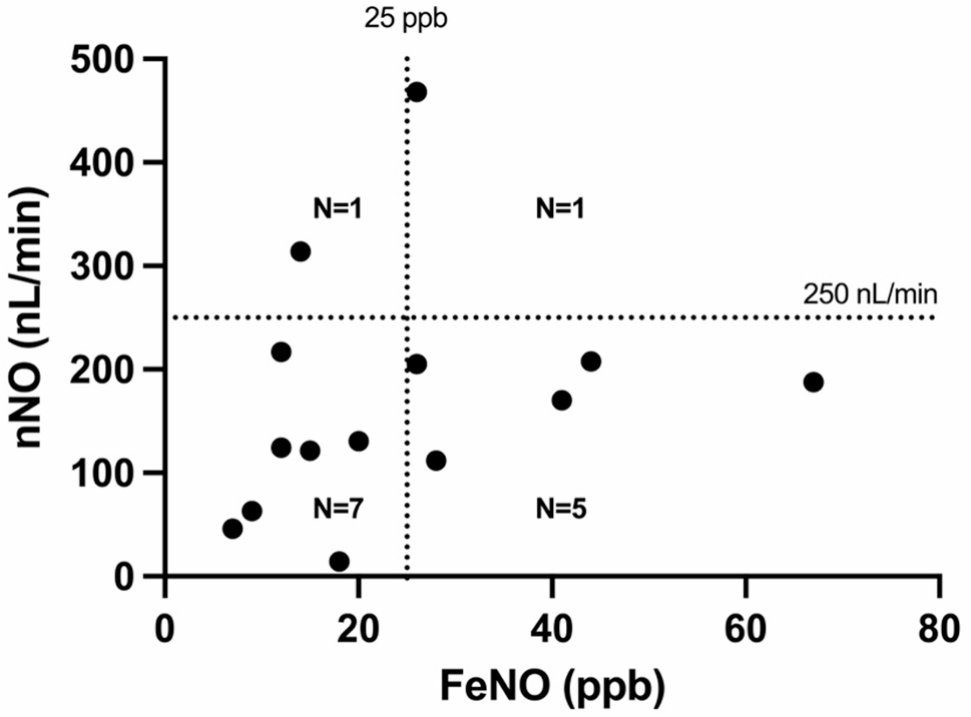
Scatter Plot of same-day nNO and FeNO measurement in 14 patients with PCD-related gene variant(s). One patient who had nNO data but unsuccessfully performed FeNO measurement did not demonstrate in this figure. The vertical dotted line marks the FeNO cut-point of 25 ppb, and the horizontal dotted line marks the normal nNO cut-point at 250 nL/min. FeNO = fractional exhaled nitric oxide; nNO = Nasal nitric oxide; PCD = Primary ciliary dyskinesia. ppb = parts per billion

nNO measurement was performed in 15 patients with PCD-related gene variants during stable state. 12 (80%) patients had low nNO values (< 250 nL/min), with 3 patients having nNO below 77 nL/min, which is the PCD screening threshold. Interestingly, comparing same-day nNO and FeNO (Fig. 3), 5 patients demonstrated discordance with the low nNO despite elevated FeNO (≥ 25 ppb). Further analysis between nNO and FeNO did not show a significant correlation (*r* = 0.35, *P* = 0.22). On separate occasions, we gathered sputum cytokine profiles obtained during symptom worsening in 4 of 5 patients. All of them evidenced intense neutrophilic bronchitis, with only one patient having concomitant uncontrolled airway eosinophilia. Not only the non-T2 cytokine (interleukin (IL)−1β, IL-18, IL-6, TNF-⍺, and BAFF) elevation, T2 inflammation signal appeared in a subset (IL-5 in 3 patients, IL-4 and IL-13 in one patient).

**Fig. 4:**
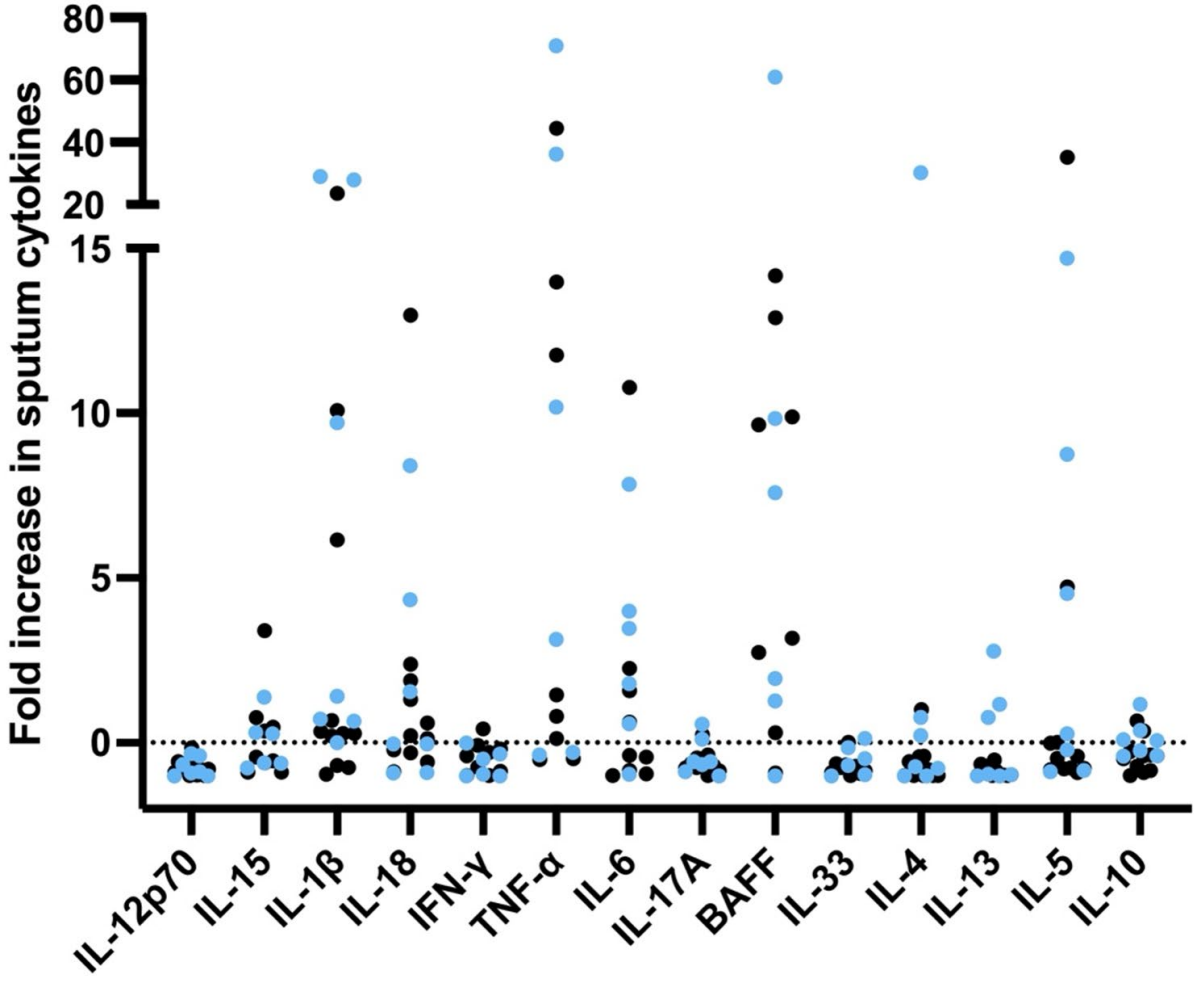
Sputum cytokine profiles in patients with PCD-related gene variant(s). Data are expressed as fold increase relative to the laboratory upper limit of normal, calculated as (cytokine concentration – upper limit of normal)/upper limit of normal; values > 0 indicate elevation. Blue dots represent patients with elevated FeNO (> 25 ppb), and black dots represent those with normal FeNO. Data were available for *N* = 18 (IL-12p70, IL-1β, IL-18, IL-17 A, IL-4, IL-13, IL-5, IL-10) and *N* = 15 (IL-15, IFN-*γ*, TNF-⍺, IL-6, BAFF, IL-33). BAFF = B-cell activating factor; FeNO = fractional exhaled nitric oxide; IL = Interleukin; IFN-γ = Interferon-gamma; PCD = Primary ciliary dyskinesia. ppb = parts per billion; TNF-α = Tumor necrosis factor-alpha

We obtained sputum cytokine profiles from 18 patients with PCD-related gene variant(s); three had incomplete panels due to limited sample volume. IL-1β, IL-18, TNF-⍺, IL-6, and BAFF represent the dominant cytokine profile, each elevated in more than 50% of patients, including those with peripheral eosinophilia and/or elevated FeNO. T2 cytokines (IL-4, IL-13, and IL-5) were elevated in 9/15(60%) patients with peripheral eosinophilia, and in 6/7(86%) patients with elevated FeNO. In the subgroup of patients with airway eosinophilia who demonstrated uncontrolled airway eosinophilia during exacerbation (*N* = 5), the dominant profile again included IL-1β, IL-18, TNF-α, IL-6, and BAFF. Among T2 cytokines, IL-5 was elevated in 2 patients and IL-13 in 1 patient. Detailed cytokine profiles are demonstrated in Table 4; Fig. 4, and e-Table 1.

**Table 4:** Sputum cytokine profiles in patients with PCD-related gene variant(s)

Cytokine	Median	IQR	Reference	% Patients Elevated
IL-12p70	0.51	0.97	2.756	0.00
IL-15	0.86	1.67	1.526	46.67
IL-1β^a^	179.50	1038.20	107.540	83.33
IL-18^a^	63.40	129.15	54.100	55.56
IFN-γ	0.07	0.10	0.126	13.33
TNF-α^a^	10.30	44.93	2.488	73.33
IL-6^a^	95.20	252.81	58.640	60.00
IL-17 A	2.51	2.45	7.068	16.67
BAFF^a^	222.00	301.20	25.880	86.67
IL-33	1.77	2.80	6.220	13.33
IL-4	0.13	0.40	0.534	22.22
IL-13	0.19	1.36	3.596	16.67
IL-5	0.34	2.66	0.494	38.89
IL-10	0.84	0.95	1.346	33.33

Data are presented as median (interquartile range, IQR) concentrations (pg/mL). Reference values were derived from the laboratory upper limit of normal. The final column shows the proportion of patients exceeding the reference value for each cytokine. Data were available for *N* = 18 (IL-12p70, IL-1β, IL-18, IL-17 A, IL-4, IL-13, IL-5, IL-10) and *N* = 15 (IL-15, IFN-, TNF-⍺, IL-6, BAFF, IL-33). BAFF = B-cell activating factor; IL = Interleukin; IFN-γ = Interferon-gamma; TNF-α = Tumor necrosis factor-alpha

^a^ Median cytokine concentration exceeded the reference value

**Fig. 5:**
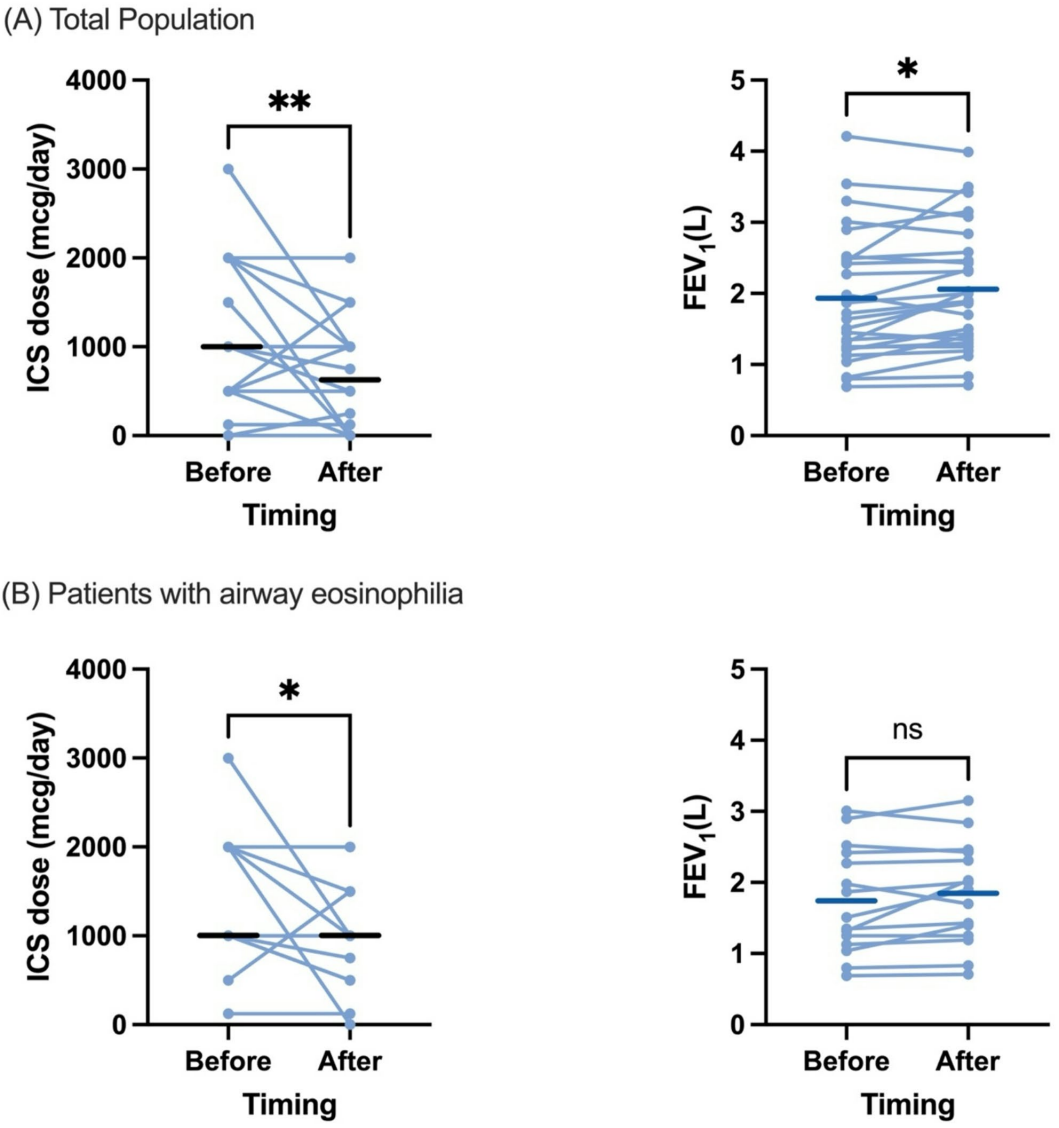
Comparison of treatment outcomes before and after treatment modification: **A** Total population (*N* = 32, with spirometry data available for 27 patients); **B** Patients with airway eosinophilia (*N* = 18, with spirometry data for 15 patients). ICS doses are expressed as fluticasone propionate equivalents. Black lines indicate the median ICS dose. Blue lines indicate the mean FEV_1_. FEV_1_ = Forced Expiratory Volume in 1 s; ICS = Inhaled Corticosteroids. *P ≤ 0.05. **P ≤ 0.01

All 32 patients in variant-positive group were prescribed 7% HTS nebulization and managed under a comprehensive severe asthma management strategy [34]. Those with neutrophilia without eosinophilia were treated with 7% HTS and antibiotics, without ICS escalation or biologics. Those with eosinophilia could receive higher ICS doses or an appropriate biologic if necessary. ICS was tapered when there was no evidence of eosinophil-driven exacerbation. This led to a significant reduction in ICS doses in 15(46.9%) patients. The ICS dose reduction was significant across the entire population and in the subgroup of patients with previous evidence of airway eosinophilia (*N* = 18), showing *P* values of 0.0026 and 0.0273, respectively. Among 27 patients with > 3 months on modified therapy, FEV_1_ increased from 1.9 ± 0.9 L to 2.1 ± 0.9 L (*P* value = 0.0273). In the eosinophilic subgroup, FEV_1_ increased by 104.7 ml (not statistically significant; *P* value = 0.1183; *N* = 15) (Fig. 5). Additionally, oral corticosteroid (OCS) doses were reduced in 6 of the 8 patients previously on them (75%). The decision to forgo biologic therapy was made for 26(81.3%) patients.

## Discussion

In our study, we highlight the significant role of ciliary dysfunction as a contributor to infection susceptibility, leading to exacerbations in adult patients with severe asthma. Through the application of our predefined clinical criteria for exome sequencing, we identified PCD-related gene variants in 61.5% of patients, accounting for 5.9% of all patients with severe asthma in our cohort. As genetic testing was performed in a clinically selected subgroup, the true prevalence of PCD-related gene variants in the overall severe asthma population may be underestimated. Although the small sample size limits the ability to pinpoint a specific clinical predictor for the presence of these PCD-related gene variants, we observed a higher prevalence of nasal polyposis among the patients with chronic rhinosinusitis in variant-positive group. Moreover, even among patients with confirmed airway eosinophilia, the prevalence of PCD-related gene variants was still high at 64.3%. This finding underscores the possibility that severe eosinophilic asthma can be exacerbated by infections, leading to neutrophil-driven process. Addressing this group of patients may require a more tailored treatment approach that considers the dual contribution of both eosinophilic and neutrophilic inflammation.

Ciliary dysfunction and its association with chronic airway diseases have been widely recognized in the past [13, 30, 35, 36]. The diagnosis of ciliary dysfunction can be approached through various methods, including electron microscopy to identify ultrastructural defects, genetic testing to detect mutations, assessing CBF using high-speed video microscopy, immunofluorescence (IF) microscopy technique [37], pulmonary radioaerosol mucociliary clearance scan [38], and measuring nNO [12]. Our study employed a comprehensive approach by combining genetic testing, CBF analysis, and nNO measurement, offering robust evidence of ciliary dysfunction [16]. Although several reports describe genotype–phenotype features of PCD in children and young adults [39–42], our integrative approach extends these observations to adults with severe asthma and confirms functional impairment even in patients with heterozygous VUS. In particular, 12 patients with VUS exhibited significant CBF abnormalities, reinforcing the importance of functional testing alongside genetic analysis. Identifying PCD-related gene variants through blood exome sequencing strengthens the likelihood that the ciliary dysfunction is due to a primary cause rather than secondary factors such as chronic infection or inflammation [43]. Our cohort also underscores the utility of genomic medicine in clinical practice for evaluating complex patients, not only in respiratory diseases [44, 45] but also in other systems [46]. The positive associations of CBF with FEV₁ and negative associations with peak sputum neutrophils further support the central role of mucociliary function in severe asthma. However, genotype–phenotype correlations in our cohort are difficult to establish. Most patients carried heterozygous VUS on a background of underlying asthma, which can independently contribute to respiratory symptoms. We observed 2 patients with a heterozygous pathogenic variant in *DNAH5*, both with recurrent pulmonary infections, and one with recurrent sinus infections. We showed reduced CBF (9.06 Hz), an immobility index of 20%, and a beating stiffly pattern in one patient, consistent with prior reports that *DNAH5* mutations cause impaired mucociliary clearance [47]. Another patient with a heterozygous VUS in *CCDC114* presented with chronic productive cough, recurrent infections, severe airflow obstruction, and reduced beat frequency (4.63 Hz) with a high immobility index (86%), consistent with the previous Volendam cohort showing moderately severe phenotypes in *CCDC114*-related PCD [42].

Low nNO is an established screening tool for PCD with high sensitivity and specificity in patients with a high pretest probability [32, 33]. A previous study showed the association of high nNO with high FeNO and increased bronchial responsiveness [48], raising the complex situation that T2 inflammation could mask ciliary dysfunction by lifting nNO values to the normal range. Our cohort is the first to examine nNO in severe asthma with ciliary dysfunction. Most patients with PCD-related gene variant(s) had subnormal nNO levels, including 5 patients with discordantly elevated FeNO. This finding supports the use of nNO as a screening tool in this specific T2-high population. Correlation analysis in 14 patients showed a non-significant correlation between nNO and FeNO (*r* = 0.35, *P* = 0.22), likely reflecting the small sample. We identified 3 patients with a combined PCD-related gene variant(s), abnormal CBF, and elevated FeNO. Each had subnormal nNO that nonetheless remained above the classic PCD cut-off, implying that residual type-2 inflammation can partially mask the nNO signal of ciliary dysfunction. Larger datasets will be needed to define an asthma-specific nNO threshold and to clarify the test’s diagnostic accuracy in the presence of concurrent T2 inflammation.

Sputum cytokine profile reflects the signal of innate immune response to infection (IL-1β, IL-18, TNF-⍺, IL-6, and BAFF). IL-1β and IL-18 were increased in 83% and 56% of patients, respectively. Both are downstream of inflammasome activation and have been implicated in severe asthma pathobiology [49]. IL-1β is also related to altered mucus composition in other airway diseases, including bronchiectasis [50] and cystic fibrosis [51], thereby worsening mucus clearance. These findings support further evaluation of targeted therapies, including the inflammasome pathway inhibitors, in this subgroup of severe asthma.

Our study did not include transmission electron microscopy for ultrastructural assessment or IF technique for identifying absent or mislocalized ciliary proteins, which could have clarified links between genotype, structural defects, and ciliary dysfunction. We also lacked a matched severe-asthma control cohort with variant-negative for direct comparison of CBF, and we did not obtain culture-based (air–liquid interface) CBF measurements to further differentiate primary from secondary ciliary defects. We used an electrochemical analyzer, which is categorized as Grade C for nNO measurement; however, this device is the most widely available in adult asthma clinics, and its use is likely to be practical and useful in future clinical settings.

In summary, this study underscores the importance of identifying ciliary motility disorders, particularly those related to PCD, in patients with severe airway disease and recurrent respiratory infections. An integrated approach combining exome sequencing, CBF analysis, and nNO enables more targeted management, helping to avoid unnecessary biologics, high-dose ICS, and burst courses of prednisone. Importantly, nNO retained screening utility in this population as most variant-positive patients had sub-normal nNO, including some with concurrently elevated FeNO. However, validation of nNO measurement obtained using electrochemical analyzers in the context of concurrent T2 inflammation remains an important knowledge gap. In addition, emerging, less technically complex adjunctive diagnosis tools, such as IF technique, may further enhance diagnostic yield and reduce the under diagnosis of PCD [52]. Although no therapy corrects the underlying genetic defect, several interventions may improve mucociliary function, including β₂-agonists, 7% HTS nebulization, and emerging agents such as the epithelial sodium channel blocker idrevloride (under investigation in PCD) [53]. Together, these strategies aim to enhance airway clearance, lower infection risk, and improve respiratory outcomes in this group of patients.

## Conclusions

Recognizing ciliary motility disorders as contributors to airway infections in adult patients with severe obstructive lung diseases can provide effective treatment and limit unnecessary use of biologics. The extended exome sequencing panels for PCD could be used as a tool. The heterozygous of PCD-related gene variant, even if labelled as a VUS, could contribute to recurrent airway infection from ciliary dysfunction, proven by CBF analysis. nNO measurement could be a useful screening test. However, the optimal cut-off for patients with T2 inflammation still needs to be established.

## Supplementary Information

Below is the link to the electronic supplementary material.

Supplementary Material 1

## Data Availability

The data are not publicly available due to patient confidentiality and institutional ethics restrictions.

## Abbreviations

BAFF: B-cell activating factor
CBF: Ciliary beat frequency
CFTR: Cystic fibrosis transmembrane conductance regulator
COPD: Chronic obstructive pulmonary disease
FeNO: Fractional exhaled nitric oxide
FEV_1_: Forced expiratory volume in 1 s
FP: Fluticasone propionate
FVC: Forced vital capacity
HTS: Hypertonic saline
ICS: Inhaled corticosteroid
IF: Immunofluorescence
IL: Interleukin
IQR: Interquartile range
LABA: Long-acting β_2_-agonist
nNO: Nasal nitric oxide
OCS: Oral corticosteroids
PCD: Primary ciliary dyskinesia
PID: Primary immunodeficiency
ppb: Parts per billion
SD: Standard deviation
T2: Type 2 inflammation
TCC: Total cell count
TNF: Tumor necrosis factor
VUS: Variant of uncertain significance
