# Supplementary material for: Unrecognized ciliary motility disorders in neutrophilic severe asthma exacerbations
Source: Allergy Asthma Clin Immunol. 2026 May 8;22:41. doi: 10.1186/s13223-026-01041-1 (PMC13321632; doi:10.1186/s13223-026-01041-1)
Supplement: Supplementary file 1 — Supplementary Material 1 [file 13223_2026_1041_MOESM1_ESM.docx]

**Supplementary Table 1** - Sputum cytokine profiles in patients with PCD-related gene variant(s) and elevated FeNO (>25 ppb), and/or blood eosinophils (≥0.3ⅹ10^9^/L).

| **No. of patient**^a^ | **Sputum cytokine concentrations (pg/mL)** | | | | | | | | | | | | | |
| --- | --- | --- | --- | --- | --- | --- | --- | --- | --- | --- | --- | --- | --- | --- |
|  | **IL-12p70** | **IL-15** | **IL-1β** | **IL-18** | **IFN-𝛾** | **TNF-⍺** | **IL-6** | **IL-17A** | **BAFF** | **IL-33** | **IL-4** | **IL-13** | **IL-5** | **IL-10** |
|  | (2.756) | (1.526) | (107.540) | (54.100) | (0.126) | (2.488) | (58.640) | (7.068) | (25.880) | (6.220) | (0.534) | (3.596) | (0.494) | (1.346) |
| 2 | 0 | *6.73* | *129* | *125* | 0 | *31.8* | *692* | 2.21 | *393* | *6.25* | 0 | 0 | *0.496* | *2.23* |
| 5 | 0.505 | NA | 26.5 | *757* | NA | NA | NA | 1.72 | NA | NA | 0.227 | 0 | 0.1 | 0.988 |
| 6 | 0 | 0.15 | *1193* | 22.1 | 0.016 | 1.21 | *95.2* | 3.77 | *276* | 0.447 | 0.308 | 0 | *2.83* | 0.847 |
| 10^b^ | 0.234 | 0.58 | *3195* | 5.46 | 0.007 | *178* | *518* | 0.911 | *222* | 0.227 | 0.148 | 0 | 0.062 | *2.93* |
| 14^b^ | 0 | 2 | *260* | *138* | 0 | *10.3* | *92.9* | 0 | *280* | 2.01 | 0 | 0 | *7.74* | 0.816 |
| 15 | 0.944 | *2.07* | 32.6 | *65.9* | 0.116 | *2.8* | 32.7 | 2.55 | *33.6* | 0.871 | 0.134 | 0.118 | 0.26 | 0.81 |
| 18^b^ | 1.88 | 0.371 | *3079* | 5.13 | 0.065 | *27.8* | 3.26 | 3.07 | *1595* | 0 | 0.125 | 0.164 | *0.633* | 1.03 |
| 19^d^ | 0.371 | *3.65* | *1151* | 52.4 | 0.126 | *91.6* | *293* | *11.1* | 0 | 5.34 | 16.5 | *7.83* | *2.73* | *1.85* |
| 21 | 0.519 | *2.25* | *145* | 37.5 | 0.032 | *46.7* | 0 | 1.64 | *505* | 2.2 | 0 | 0.306 | *18* | 0.373 |
| 22^d^ | 1.71 | 0.598 | *186* | 52.5 | 0.083 | 1.59 | *164* | 2.45 | *76.4* | 3.25 | 0.654 | 0.208 | 0.085 | *1.48* |
| 23^b^ | 0.962 | NA | *179* | *508* | NA | NA | NA | *7.86* | NA | NA | 0.948 | *6.38* | *4.81* | *1.44* |
| 25 | 2.34 | 0.86 | 3.81 | 6.56 | *0.178* | 1.29 | 2.84 | *8.73* | 2.12 | 0 | 1.07 | 1.7 | 0.291 | *1.83* |
| 30 | 0.657 | NA | *180* | *60.9* | NA | NA | NA | 0.972 | NA | NA | 0.096 | 0.514 | 0.117 | 0.204 |
| 31 | 0.682 | 0.156 | *2675* | *183* | 0 | *114* | *191* | 3.61 | *108* | 2.02 | 0.313 | 0.048 | 0.105 | 0.694 |
| 32^c^ | 0 | *1.96* | *108* | *289* | 0 | 1.77 | *262* | 3.12 | *58.8* | *7.1* | 0 | *13.6* | 0.393 | 0.833 |

Data are sputum supernatant cytokine concentrations (pg/mL); undetectable values are shown as 0. Laboratory upper limits of normal are shown in parentheses. Values presented in *italic* indicate concentrations above the upper limit of normal. 7 patients have elevated FeNO.
BAFF = B-cell activating factor; FeNO = fractional exhaled nitric oxide; IL = Interleukin; IFN-γ = Interferon-gamma; nNO = Nasal nitric oxide; PCD = Primary ciliary dyskinesia. ppb = parts per billion. TNF-α = Tumor necrosis factor-alpha.

^a^ Referred to the number of patients with PCD-related gene variant(s) in Table 2.

^b^ Patients with elevated FeNO (>25 ppb) with nNO < 250 nL/min.

^c^ Patient with elevated FeNO (>25 ppb) with nNO ≥ 250 nL/min.

^d^ Patients with elevated FeNO (>25 ppb) but no nNO data available.
